# Work Stress, Quality of Work Life, and Burnout Among Registered Nurses in Public Hospitals in Samut Songkhram Province, Thailand: An Explanatory Sequential Mixed-Methods Study

**DOI:** 10.3390/ijerph23080961

**Published:** 2026-07-25

**Authors:** Bovornpot Choompunuch, Emanyah Sani, Jutatip Sillabutra, Jatuporn Ounprasertsuk

**Affiliations:** 1 Department of Educational Psychology and Guidance, Faculty of Education, Mahasarakham University, Maha Sarakham 44000, Thailand; bovornpot.c@msu.ac.th; 2 Department of Medical and Public Health Secretary, College of Allied Health Sciences, Suan Sunandha Rajabhat University, Samut Songkhram 75000, Thailand; s66122236013@ssru.ac.th; 3 Department of Biostatistics, Faculty of Public Health, Mahidol University, Bangkok 10400, Thailand; jutatip.sil@mahidol.ac.th

**Keywords:** burnout, registered nurses, work stress, occupational stress, quality of work life, public hospitals, mixed methods, Thailand

## Abstract

**Highlights:**

**Public health relevance—How does this work relate to a public health issue?**

- Nurse burnout is an occupational and public health issue that may affect workforce well-being, retention, and the quality and safety of care in public hospitals.
- This study addresses burnout among registered nurses in Thai public hospitals by examining work stress, quality of work life, and nurses’ lived workplace experiences.

**Public health significance—Why is this work of significance to public health?**

- The findings show that work stress and quality of work life jointly explained 35.0% of the variance in burnout among registered nurses.
- Work–life balance, fair compensation, professional development, workplace safety, organizational justice, and social integration were key modifiable workplace factors associated with lower burnout scores.

**Public health implications—What are the key implications or messages for practitioners, policy makers and/or researchers in public health?**

- Hospital administrators and nurse managers should prioritize organizational strategies that improve work–life balance, staffing support, fair compensation, and a supportive organizational climate.
- Occupational health nursing interventions may move beyond individual stress management and address workplace-level conditions that contribute to burnout and nurse retention.

**Abstract:**

Burnout among nurses is an important occupational and public health concern because it is associated with nurses’ well-being, workforce retention, and the quality and safety of patient care. This explanatory sequential mixed-methods study examined levels of work stress, quality of work life, and burnout among registered nurses in public hospitals in Samut Songkhram Province, Thailand, and explored nurses’ experiences regarding workplace factors related to burnout. The target quantitative sample was 217 registered nurses, and 186 completed questionnaires were included in the final analysis. Structured questionnaires assessed work stress, quality of work life, and burnout. Multiple regression analysis was used to identify factors associated with burnout. Qualitative data were collected through in-depth interviews with 10 registered nurses and analyzed using content analysis. Most nurses were classified as having high work stress and being at risk of burnout. Work stress and quality of work life jointly explained 35.0% of the variance in burnout. Work–life balance, fair and adequate compensation, professional development, workplace safety, organizational justice, and social integration in the workplace were associated with lower burnout scores. Organizational structure, policy, and climate showed a positive coefficient in relation to burnout and should be interpreted cautiously because the *p*-value was close to the conventional threshold for statistical significance. Qualitative findings supported the quantitative results and highlighted workload, staffing shortages, limited resources, insufficient recovery time, and workplace support as important workplace concerns.

## 1. Introduction

Burnout among nurses has become a persistent occupational health and public health issue because nursing work is characterized by high emotional demands, complex patient care responsibilities, shift work, time pressure, and continuous exposure to human suffering. Recent systematic reviews and meta-analyses have shown that nurse burnout is not only a problem of individual distress but is also associated with lower healthcare quality, reduced patient safety, and lower patient satisfaction [1]. Burnout is commonly reflected through emotional exhaustion, depersonalization or cynicism, and reduced professional efficacy, and these dimensions are especially relevant to hospital nursing, where nurses must maintain clinical vigilance and emotional engagement under sustained workload pressure [1,2]. Evidence also suggests that burnout contributes to organizational and position turnover among nurses, making it a workforce sustainability issue for health systems facing staffing shortages and increasing service demand [3].

Work stress is a central antecedent of burnout in nursing because it reflects the degree to which job demands exceed the resources available to meet those demands. A review of key job demands and resources among nursing staff identified workload, work–home conflict, emotional demands, and staffing-related pressures as major job demands, while social support, leadership, professional development, and autonomy functioned as important job resources [4]. These findings are consistent with the Job Demands–Resources perspective, which suggests that burnout is more likely to occur when high job demands are not balanced by sufficient workplace resources. In hospital settings, organizational culture and climate are particularly important because policies, managerial practices, communication patterns, and team relationships can either buffer or intensify work-related stress among nurses [5].

Quality of work life is increasingly recognized as a modifiable workplace resource that can protect nurses from burnout. It includes nurses’ perceptions of fair compensation, safe working conditions, career development, social integration, organizational justice, and balance between work and personal life. A systematic review reported that nurses frequently experience moderate to high burnout and that burnout is negatively associated with quality of life, supporting the need for organizational interventions that improve nurses’ working conditions and well-being [6]. Recent evidence also indicates that workload is inversely associated with nurses’ quality of work life, meaning that heavier workload tends to reduce perceived work–life quality and may increase vulnerability to stress and burnout [7]. Work–life balance is especially important in nursing because shift work, overtime, and high workload can limit recovery time, disrupt family and personal life, and undermine psychological well-being [8].

The Job Demands–Resources model provides a useful framework for understanding burnout in this study. From this perspective, work stress reflects job demands, such as workload, emotional demands, time pressure, and organizational constraints, whereas quality of work life reflects job resources, such as fair compensation, safe working conditions, social integration, organizational justice, work–life balance, and opportunities for professional development [4,9]. Burnout is more likely to occur when job demands remain high and are not balanced by sufficient job resources, and recent nursing studies have applied the Job Demands–Resources model to explain how workplace demands and resources are associated with nurse burnout and work-related outcomes [9,10]. Applying this framework to public hospital nursing is important because nurses’ experiences of burnout are shaped not only by individual coping capacity but also by the organizational environment in which care is delivered [5,11].

Public hospitals in provincial Thailand are important settings for examining this issue because they provide essential healthcare services to local populations while operating within workforce and resource constraints. Previous studies in Thailand have documented burnout and work-related stress among healthcare workers, but less is known about how work stress and quality of work life interact in provincial public hospital nursing contexts [12,13,14]. Samut Songkhram Province is a small provincial health service context in central Thailand where public hospitals serve both local residents and surrounding communities. Studying registered nurses in this setting allows the present study to examine burnout in a real-world provincial public hospital context, where staffing patterns, extended working hours, and organizational policies may shape nurses’ work stress and quality of work life. This setting is therefore relevant for generating practical evidence for occupational health nursing and public hospital workforce management in Thailand.

Therefore, this study aimed to examine levels of work stress, quality of work life, and burnout among registered nurses in public hospitals in Samut Songkhram Province, Thailand; identify dimensions of work stress and quality of work life associated with burnout; and explore nurses’ experiences of workplace factors related to stress, quality of work life, and burnout. The study was guided by the following research questions: (1) What are the levels of work stress, quality of work life, and burnout among registered nurses in public hospitals? (2) Which dimensions of work stress and quality of work life are associated with burnout? (3) How do registered nurses describe workplace factors that contribute to or reduce burnout in their daily practice? (4) How do the qualitative findings explain and expand the quantitative results? By integrating quantitative and qualitative evidence, this study contributes to occupational health nursing by identifying modifiable workplace resources that may help hospital administrators and nurse managers design strategies to reduce burnout, improve quality of work life, and support nurse retention in public healthcare settings.

## 2. Materials and Methods

### 2.1. Study Design

This study employed an explanatory sequential mixed-methods design, in which quantitative data are collected and analyzed first and then used to inform qualitative data collection and interpretation [15]. The manuscript was reported with reference to the Good Reporting of A Mixed Methods Study (GRAMMS) framework for mixed-methods research and relevant STROBE guidance for the quantitative cross-sectional phase [16,17]. The quantitative phase was conducted first using a cross-sectional survey to examine levels of work stress, quality of work life, and burnout among registered nurses, as well as to identify dimensions of work stress and quality of work life associated with burnout. The qualitative phase was then conducted through in-depth interviews to explain, expand, and contextualize the quantitative findings. This design was selected because burnout is a multidimensional occupational phenomenon that can be measured statistically while also requiring an understanding of nurses’ lived experiences within their workplace context.

The transition from the quantitative to the qualitative phase was based on preliminary analysis of the survey findings. After identifying key patterns in work stress, quality of work life, and burnout, the researchers purposively selected nurses who could provide detailed explanations of these patterns. Selection considered direct nursing experience, department or unit, working-hour patterns, and burnout-related experiences. The qualitative phase was intended to explain and expand the quantitative findings rather than to produce statistical generalization.

### 2.2. Study Setting and Participants

The study was conducted in public hospitals in Samut Songkhram Province, Thailand. These hospitals provide essential healthcare services to the local population and nearby areas and include nursing units such as outpatient departments, inpatient departments, emergency and operating units, labor rooms, intensive care units, and administrative nursing units.

The target population consisted of registered nurses working in public hospitals in Samut Songkhram Province. According to hospital workforce records, the total population included 340 registered nurses, comprising 280 nurses from Somdet Phra Phutthaloetla Hospital and 60 nurses from Amphawa Hospital. The quantitative sample size was calculated using the Krejcie and Morgan formula [18], resulting in a required sample of 217 nurses. A total of 186 completed questionnaires were returned and included in the final analysis, representing 85.7% of the required sample and 54.7% of the eligible population. The final analyzed sample was lower than the calculated sample size because not all invited nurses returned completed questionnaires during the data collection period. Returned questionnaires were screened for completeness before data entry, and questionnaires with substantial missing data or incomplete responses on key study variables were excluded from the final analysis. For the planned multiple regression analysis, the model included 12 predictors, resulting in approximately 15.5 participants per predictor, which exceeded commonly used minimum recommendations for multiple regression.

For the qualitative phase, 10 registered nurses were purposively selected from those who had relevant work experience and were able to provide rich information regarding work stress, quality of work life, and burnout. Selection aimed to capture meaningful perspectives related to the quantitative results and the real-world working conditions of nurses in public hospital settings.

### 2.3. Eligibility Criteria

For the quantitative phase, eligible participants were registered nurses working in public hospitals in Samut Songkhram Province, had at least one year of work experience, were able to read, write, and communicate in Thai, and voluntarily agreed to participate by providing written informed consent. Nurses working in head nurse or nursing administration roles were eligible only if they retained direct nursing practice, supervisory, or unit-based clinical responsibilities. Nurses holding senior administrative positions without direct nursing practice or unit-based clinical responsibilities were excluded. Participants were also excluded if they were on study leave, maternity leave, sick leave, or other forms of leave during the data collection period, withdrew from the study, or returned questionnaires with incomplete data that did not meet the criteria for analysis.

For the qualitative phase, 10 registered nurses were purposively selected after preliminary quantitative analysis. Participants were selected based on their direct nursing experience, ability to provide detailed information about workplace stress and burnout, and variation in department or unit, working-hour patterns, and years of work experience. Although participants were not selected to statistically represent each burnout category, the selection was guided by the need to explain and expand the quantitative findings. Interviews continued until no substantially new themes emerged, indicating that thematic saturation was sufficient for the explanatory purpose of the qualitative phase.

### 2.4. Quantitative Instruments

The quantitative questionnaire consisted of four sections. The first section assessed participants’ demographic and work-related characteristics, including sex, age, marital status, educational level, job position, income, department or unit, years of government service, and average working hours per day.

The second section assessed work stress using an adapted Thai version of the Occupational Stress Indicator, originally developed to assess sources and consequences of occupational stress in organizational settings [19]. The scale included 40 items across four dimensions: job characteristics, career achievement and advancement, interpersonal relationships, and organizational structure, policy, and climate. Responses were rated on a 5-point scale from 1 to 5, with higher scores indicating greater perceived work stress. Mean scores were interpreted as low (1.00–2.33), moderate (2.34–3.67), or high (3.68–5.00). The adapted work stress scale was reviewed by three experts in nursing research, public health administration, and research methodology to assess content validity, clarity, and contextual appropriateness for the Thai public hospital nursing context. Items with an item-level content validity index (I-CVI) of 1.00 were retained, while items with lower values were revised according to expert feedback. The scale-level content validity index based on the average method (S-CVI/Ave) was 0.94, indicating acceptable content validity. The instrument was then pilot-tested with registered nurses who had characteristics similar to the study participants. In the present study, the Cronbach’s alpha coefficient for the overall work stress scale was 0.92, and the dimension-specific Cronbach’s alpha coefficients ranged from 0.90 to 0.94.

The third section assessed quality of work life using a Thai questionnaire developed and adapted from Walton’s conceptual framework [20]. The scale included 20 items across eight dimensions: fair and adequate compensation, safe and healthy working conditions, career security and advancement, opportunities for knowledge and skill development, social integration in the workplace, fairness and respect for individual rights, work–life balance, and social responsibility. Responses were rated on a five-point scale; mean scores were interpreted as very poor (1.00–1.80), poor (1.81–2.60), moderate (2.61–3.40), good (3.41–4.20), or very good (4.21–5.00), with higher scores indicating better perceived quality of work life. The quality of work life questionnaire was reviewed by three experts in nursing research, public health administration, and research methodology to assess content validity, clarity, and contextual appropriateness for registered nurses in Thai public hospitals. Items with an I-CVI of 1.00 were retained, while items with lower values were revised according to expert feedback. The S-CVI/Ave was 0.92, indicating acceptable content validity. The questionnaire was then pilot-tested with registered nurses who had characteristics similar to the study participants. In the present study, the Cronbach’s alpha coefficient for the overall quality of work life scale was 0.90, and the dimension-specific Cronbach’s alpha coefficients ranged from 0.89 to 0.92.

The fourth section assessed burnout using a Thai version adapted from the Maslach Burnout Inventory–General Survey [21]. The scale included 16 items across three dimensions: emotional exhaustion, depersonalization or cynicism, and reduced personal accomplishment. Items were rated on a seven-point frequency scale ranging from 0 to 6. Mean scores were interpreted as no burnout risk (0.00–1.20), low burnout risk (1.21–2.40), moderate burnout risk (2.41–3.60), at risk of burnout (3.61–4.80), or burnout (4.81–6.00). Reverse-scored burnout items were recoded before calculating dimension and overall scores so that higher scores consistently indicated greater burnout risk. The adapted burnout scale was reviewed by three experts in nursing research, public health administration, and research methodology to assess content validity, clarity, and contextual appropriateness for the Thai public hospital nursing context. Items with an I-CVI of 1.00 were retained, while items with lower values were revised according to expert feedback. The S-CVI/Ave was 0.94, indicating acceptable content validity. The instrument was then pilot-tested with registered nurses who had characteristics similar to the study participants. In the present study, the Cronbach’s alpha coefficient for the overall burnout scale was 0.92, and the dimension-specific Cronbach’s alpha coefficients ranged from 0.90 to 0.93.

### 2.5. Qualitative Instrument

The semi-structured interview guide was developed based on the study objectives, literature review, Job Demands–Resources framework, and preliminary quantitative findings. The quantitative results informed the interview topics by identifying areas requiring further explanation, including workload, work–life balance, organizational climate, social support, professional development, and burnout experiences. The guide was reviewed by experts in nursing research and public health administration to assess clarity, relevance, and alignment with the explanatory sequential design. The guide was refined before data collection.

Sample interview questions included: “*Can you describe situations in your work that make you feel stressed or emotionally exhausted?*”; “*How do your working hours or shift patterns affect your personal life and recovery time?*”; “*What kinds of support from supervisors, colleagues, or the organization help you manage stress?*”; and “*What workplace changes do you think would improve nurses’ quality of work life and reduce burnout risk?*”

The interview format allowed participants to describe their experiences freely while enabling the interviewer to probe for additional detail when needed. Each interview lasted approximately 45–60 min and was audio-recorded with participants’ permission. Interviews continued until the research team determined that no substantially new themes were emerging from the data. The final sample of 10 nurses was considered sufficient for the explanatory purpose of the qualitative phase because the interviews provided repeated and consistent accounts of workload, work–life imbalance, organizational support, and professional development.

### 2.6. Data Collection

Data were collected between 15 January and 28 February 2026. Quantitative data were collected after approval was obtained from the relevant ethics committee and permission was granted by the participating hospitals. The researchers coordinated with nursing administrators and unit leaders to explain the study objectives, procedures, participant rights, and data collection process.

Eligible registered nurses were invited to complete a self-administered questionnaire. Before participation, the researchers explained the purpose of the study, confidentiality procedures, voluntary participation, and the right to withdraw at any time without consequences. Participants who agreed to participate provided written informed consent. The questionnaire required approximately 20–30 min to complete. Returned questionnaires were reviewed for completeness before data entry.

After preliminary quantitative analysis, qualitative data were collected through face-to-face in-depth interviews. Participants were selected purposively to provide deeper insight into the patterns observed in the quantitative phase. Interviews were conducted at a time and place convenient for participants. Before each interview, the researcher explained the purpose of the qualitative phase, confidentiality protection, the voluntary nature of participation, and the right to stop the interview at any time. With permission, interviews were audio-recorded and later transcribed verbatim for analysis.

### 2.7. Data Analysis

Quantitative data were analyzed using IBM SPSS Statistics version 29 (IBM Corp., Armonk, NY, USA). Descriptive statistics were used to summarize participants’ demographic and work-related characteristics. Frequencies and percentages were used for categorical variables, while means and standard deviations were used for continuous variables and scale scores. Levels of work stress, quality of work life, and burnout were interpreted according to the scoring criteria of each instrument. Standard multiple linear regression using simultaneous entry was performed to examine the extent to which dimensions of work stress and quality of work life were associated with burnout. The dependent variable was the overall burnout score, and the independent variables included four dimensions of work stress and eight dimensions of quality of work life. The overall burnout score was used to provide a parsimonious summary of burnout risk and to align with the study objective of examining overall burnout among registered nurses. However, burnout is a multidimensional construct, and the use of an aggregate score may obscure dimension-specific associations.

Demographic and work-related characteristics were summarized descriptively but were not included as covariates in the primary regression model. The primary purpose of the regression analysis was to examine the specific contributions of work stress and quality of work life dimensions to burnout. Some demographic variables, including sex and educational level, also had highly unbalanced categories, which limited their suitability for inclusion as covariates. The absence of demographic adjustment was therefore acknowledged as a limitation and should be considered when interpreting the findings. Before conducting multiple regression analysis, key assumptions were examined. Normality of residuals was assessed using visual inspection of histograms and normal probability plots. Multicollinearity was evaluated using tolerance and variance inflation factor values. Independence of errors was assessed using the Durbin–Watson statistic. Scatterplots of standardized residuals and predicted values were inspected to assess linearity and homoscedasticity. Influential observations were assessed using standardized residuals and influence diagnostics. No serious violations were identified that would preclude interpretation of the regression model. Statistical significance was set at *p* < 0.05.

Qualitative data were analyzed using directed content analysis informed by the quantitative findings and the Job Demands–Resources framework, while allowing new categories to emerge from participants’ accounts. Interview transcripts were read repeatedly to gain familiarity with the data. Meaningful units related to work stress, quality of work life, workplace resources, and burnout experiences were identified and coded. Codes were then grouped into categories and themes through discussion among the research team. The final themes were compared with the quantitative findings to determine how the qualitative data explained, expanded, or contextualized the statistical results.

### 2.8. Trustworthiness of Qualitative Data

Several strategies were used to enhance the trustworthiness of the qualitative findings. Credibility was supported by using open-ended questions, probing participants’ responses, and comparing qualitative themes with the quantitative findings. Dependability was enhanced through consistent interview procedures, systematic coding, and documentation of analytic decisions. Confirmability was supported by maintaining an audit trail that included interview notes, coding decisions, theme development, and discussion of coding interpretations among the research team. Transferability was addressed by providing contextual information about the public hospital setting, nursing units, participant characteristics, and work experiences.

## 3. Results

### 3.1. Participant Characteristics

A total of 186 registered nurses completed the quantitative survey and were included in the final analysis. Most participants were female (99.46%), and approximately one-third were aged 21–30 years (33.87%). Nearly half were married or living with a spouse (47.85%), and most held a bachelor’s degree (97.85%). Regarding professional position, more than half were professional nurses at the practitioner level (58.06%), followed by registered nurses at the operational level (31.18%). The largest proportion of participants worked in inpatient departments (42.47%), followed by specialized units such as intensive care and coronary care units (23.12%). More than two-thirds of participants reported working more than 10 h per day (71.51%). Participant characteristics are presented in Table 1.

### 3.2. Levels of Work Stress, Quality of Work Life, and Burnout

Table 2 presents the overall categorical levels of work stress, quality of work life, and burnout. Most registered nurses reported a high level of work stress (66.13%), while the remaining participants reported a moderate level of work stress (33.87%). Regarding quality of work life, the largest proportion of participants reported a good level (39.78%), followed by very good and moderate levels in equal proportions (26.34% each). For burnout, most participants were classified as being at risk of burnout (60.75%), followed by moderate burnout risk (26.34%). Only 1.08% of participants were classified as having no burnout risk, while 2.69% were classified as experiencing burnout.

### 3.3. Mean Scores of Work Stress, Quality of Work Life, and Burnout

The mean scores for each dimension of work stress, quality of work life, and burnout are shown in Table 3. The overall mean score for work stress was 3.54 (SD = 0.52), indicating a moderate level of work stress. Among the work stress dimensions, interpersonal relationships had the highest mean score (M = 3.62, SD = 0.56), followed by job characteristics (M = 3.53, SD = 0.48).

Although 66.13% of participants were classified as having high work stress based on individual-level categorical scoring, the overall mean score for work stress was interpreted as moderate. This difference reflects the use of two different summary approaches. The categorical result describes the distribution of individual participants across score ranges, whereas the mean score represents the average level of work stress across all participants and dimensions. Therefore, the results suggest that high work stress was common at the individual level, even though the overall average score remained within the moderate range.

The overall mean score for quality of work life was 4.07 (SD = 0.61), indicating a high level. The highest mean score was observed for social responsibility (M = 4.21, SD = 0.71), followed by fairness and respect for individual rights (M = 4.07, SD = 0.70) and opportunities for knowledge and skill development (M = 4.00, SD = 0.71). The overall mean score for burnout was 3.67 (SD = 1.22), indicating that participants were generally at risk of burnout. Depersonalization or cynicism had the highest mean score (M = 4.43, SD = 1.16), followed by emotional exhaustion (M = 4.23, SD = 1.36).

### 3.4. Predictors of Burnout

Multiple regression analysis was conducted to examine whether dimensions of work stress and quality of work life were associated with burnout among registered nurses (Table 4). The overall model was statistically significant, F(12, 173) = 7.76, *p* < 0.001, and explained 35.0% of the variance in burnout, with an adjusted R^2^ of 0.305. The regression model included four dimensions of work stress and eight dimensions of quality of work life. Demographic and work-related characteristics were not included as covariates in this primary model. Therefore, the findings should be interpreted as associations between workplace psychosocial factors and burnout before adjustment for demographic characteristics.

In the multivariable regression model, six quality of work life dimensions were significantly associated with lower burnout scores. Work–life balance showed the strongest negative association with burnout (β = −0.35, *p* = 0.002), followed by fair and adequate compensation (β = −0.31, *p* = 0.004), opportunities for knowledge and skill development (β = −0.27, *p* = 0.02), fairness and respect for individual rights (β = −0.26, *p* = 0.02), safe and healthy working conditions (β = −0.22, *p* = 0.03), and social integration in the workplace (β = −0.18, *p* = 0.04). Career security and advancement and social responsibility were not statistically significant predictors of burnout in the multivariable model.

Among the work stress dimensions, organizational structure, policy, and climate were positively associated with burnout (β = 0.23, *p* = 0.04). Interpersonal relationships showed a negative coefficient (β = −0.24, *p* = 0.04); however, this unexpected finding should be interpreted cautiously because it may reflect shared variance with other work stress and quality of work life dimensions, possible suppression effects, or overlap between interpersonal stress and social integration in the workplace. This finding should not be interpreted as evidence that interpersonal stress reduces burnout.

### 3.5. Qualitative Findings

The qualitative phase provided additional insight into nurses’ experiences of work stress, quality of work life, and burnout (Table 5). Content analysis identified four main themes: workload and increased responsibilities, work–life imbalance, organizational and peer support, and professional development and self-care. These themes explained and expanded the quantitative findings by showing how workplace demands and resources shaped nurses’ burnout experiences in daily practice.

#### 3.5.1. Workload and Increased Responsibilities

Participants described heavy workload, staffing shortages, high patient volume, and responsibility for urgent clinical situations as major sources of stress. These conditions often required nurses to continue working despite fatigue and limited opportunities for rest during shifts. One participant stated, “When there are many patients but not enough nurses, we have to continue working even when we are already tired. Sometimes there is no real time to rest during the shift” (Participant 3, inpatient unit). This theme supports the quantitative finding that many nurses were classified as having high work stress and being at risk of burnout.

#### 3.5.2. Work–Life Imbalance

Work–life imbalance was also described as an important contributor to stress and burnout, particularly among nurses working rotating shifts or overtime. Participants reported that extended working hours reduced time for family, personal recovery, and self-care. One participant explained, “After working overtime or rotating shifts, I often feel that I do not have enough time for my family or for myself. The tiredness continues even after I go home” (Participant 6, specialized unit). This finding helps explain why work–life balance showed the strongest negative association with burnout scores in the regression model.

#### 3.5.3. Organizational and Peer Support

Participants emphasized that support from supervisors, colleagues, and the organization helped them manage stress and maintain morale. A supportive team environment, fair treatment, and open communication were viewed as important resources in daily nursing practice. One participant noted, “Support from colleagues and supervisors helps a lot. When the team helps each other and the head nurse listens, the stress becomes easier to manage” (Participant 2, outpatient department). This theme supports the quantitative findings related to social integration, organizational justice, and organizational climate.

#### 3.5.4. Professional Development and Self-Care

Participants also described professional development and self-care as important coping resources. Opportunities for training helped nurses feel more confident when caring for patients with complex needs, while rest, exercise, family time, and peer consultation helped them manage stress. One participant stated, “Training makes me feel more confident when taking care of complicated patients. I also try to rest, exercise, and talk with colleagues when I feel stressed” (Participant 8, emergency/operating unit). This theme helps explain why opportunities for knowledge and skill development were associated with lower burnout scores.

### 3.6. Integration of Quantitative and Qualitative Findings

The integration of quantitative and qualitative findings showed how the qualitative phase explained and expanded the statistical findings. Quantitatively, work stress and quality of work life dimensions jointly explained 35.0% of the variance in burnout. Qualitatively, nurses described how these associations were experienced in practice: workload, staffing shortages, extended working hours, and organizational constraints created sustained demands, whereas work–life balance, fair treatment, peer and supervisor support, safe working conditions, and opportunities for professional development were described as workplace resources that helped nurses manage stress. The qualitative findings therefore added contextual explanation by showing how nurses experienced the organizational conditions represented by the survey variables. Table 6 presents a joint display of the integrated findings.

## 4. Discussion

This explanatory sequential mixed-methods study examined work stress, quality of work life, and burnout among registered nurses working in public hospitals in Samut Songkhram Province, Thailand. The findings showed that most nurses reported high work stress and were at risk of burnout, while the overall quality of work life was rated at a high level. Work stress and quality of work life jointly explained 35.0% of the variance in burnout, indicating that burnout was not only related to individual perceptions of stress but also shaped by modifiable workplace conditions. The qualitative findings further clarified that workload, staffing shortages, extended working hours, limited resources, work–life imbalance, and organizational support were central to nurses’ experiences of stress and burnout. These integrated findings support the view that nurse burnout should be understood as an occupational and organizational phenomenon rather than solely as an individual psychological response [2,3]. Both our quantitative and qualitative results also strengthen the occupational health relevance of the study because they identify workplace resources that may be modified by hospital administrators and nurse managers to reduce burnout risk. Recent reviews similarly emphasize that nurse burnout affects not only nurses’ well-being but also patient safety, quality of care, patient satisfaction, productivity, and workforce retention [1,4].

The high proportion of nurses reporting work stress and burnout risk is consistent with global evidence showing that nursing remains a high-demand profession characterized by emotional labor, shift work, staffing pressure, and continuous responsibility for patient safety [4,22]. The overall burnout score in this study suggested that participants were generally at risk of burnout, with depersonalization or cynicism and emotional exhaustion showing particularly high mean scores. This pattern is important because emotional exhaustion reflects depletion of psychological and physical energy, while depersonalization may indicate emotional distancing from patients as a coping response to sustained work pressure [23,24]. Previous systematic reviews have shown that burnout among nurses is associated with poorer patient safety climate, lower quality of care, reduced patient satisfaction, and adverse organizational outcomes [3,25]. Therefore, the present findings suggest that burnout among nurses in provincial public hospitals should be considered a workforce sustainability issue, not only a mental health concern.

The findings can be interpreted through the Job Demands–Resources framework, which proposes that burnout develops when job demands remain high and are not balanced by adequate job resources [4,9]. In the present study, nurses described heavy workload, staffing shortages, long working hours, and resource constraints as major sources of stress. These factors are typical job demands in hospital nursing and may increase emotional exhaustion when nurses are required to maintain clinical accuracy and emotional responsiveness under time pressure. Similar evidence has identified workload, work–home conflict, emotional demands, and staffing-related pressure as key job demands among nursing staff [4]. Studies on nurse work environments also show that inadequate staffing and high workload are associated with burnout and poorer outcomes for nurses and patients [10,22]. The qualitative findings of this study add depth to these quantitative patterns by showing that stress was experienced not only as “more work,” but also as insufficient recovery time, limited control over schedules, and pressure to maintain care quality despite resource constraints.

Quality of work life was a central workplace resource associated with lower burnout scores in this study. Several dimensions of quality of work life were negatively associated with burnout, including work–life balance, fair and adequate compensation, opportunities for professional development, safe and healthy working conditions, fairness and respect for individual rights, and social integration in the workplace. These findings are consistent with evidence that nurses’ burnout is negatively associated with quality of life and that improving workplace conditions is essential for reducing burnout [6,11]. In this study, quality of work life functioned as a set of job resources that could buffer the effects of work stress. This finding is particularly important for occupational health nursing because it shifts the intervention focus from individual coping alone to organizational conditions that can be changed, monitored, and improved. Recent intervention reviews also suggest that workplace-level strategies, leadership support, workload redesign, and organizational interventions may be necessary alongside individual-level stress management approaches [26,27].

Work–life balance had the strongest negative association with burnout. This finding is highly plausible in the nursing context, where rotating shifts, overtime, night duty, and unpredictable workload can interfere with sleep, family responsibilities, personal recovery, and psychological detachment from work. Recent studies have reported that poor work–life balance is associated with worse mental health, lower work engagement, and higher burnout among nurses [8,28,29]. The qualitative findings in this study supported this result, as nurses described limited time for rest, family, and personal activities as a major contributor to cumulative fatigue. This suggests that hospital strategies to reduce burnout should include practical scheduling reforms, adequate rest periods, fair distribution of overtime, and staffing plans that reduce excessive work hours. Work–life balance should not be framed as a personal responsibility alone; it is also an organizational outcome shaped by staffing policy and managerial decisions.

Fair and adequate compensation was another quality of work life dimension associated with lower burnout scores. This finding aligns with effort–reward imbalance theory and recent nursing studies showing that perceived imbalance between effort and reward is associated with higher burnout and poorer well-being [30,31,32]. For nurses, compensation is not only an economic issue but also a signal of organizational recognition and fairness. When compensation is perceived as insufficient relative to workload, risk, responsibility, and emotional labor, nurses may feel undervalued, which can intensify exhaustion and disengagement. The qualitative findings also suggested that nurses’ concerns about workload and organizational support were closely tied to perceptions of fairness. Therefore, compensation policy, workload-based incentives, and recognition systems should be considered part of burnout prevention and nurse retention strategies.

Organizational structure, policy, and climate showed a positive coefficient in relation to burnout, suggesting that organizational context may be relevant to nurses’ psychological well-being. This interpretation is consistent with evidence linking organizational culture, organizational climate, leadership, and workplace support with nurses’ work-related stress, burnout, and well-being [5,33,34]. However, this finding should be interpreted cautiously because the *p*-value was close to the conventional threshold for statistical significance.

The negative association between social integration in the workplace and burnout reinforces the importance of collegial support, teamwork, and belonging in nursing practice. Nurses in the qualitative phase described support from supervisors and colleagues as a key resource that helped them manage work pressure, maintain morale, and cope with difficult clinical situations. This interpretation is consistent with evidence that perceived organizational support, social support, and positive workplace relationships are associated with lower burnout and better occupational well-being among nurses [35,36]. In contrast, poor interpersonal relationships, workplace conflict, bullying, or lack of trust may intensify emotional exhaustion and depersonalization [37,38]. Therefore, hospital administrators and nurse managers should consider supportive supervision, peer support systems, team-based interventions, and clear mechanisms for addressing workplace conflict as part of broader burnout prevention strategies.

Opportunities for knowledge and skill development were also negatively associated with burnout. This suggests that professional development may serve as a psychological and occupational resource by increasing competence, confidence, professional identity, and perceived career growth. Nurses in the qualitative phase reported that continuing development helped them feel more prepared to manage complex situations and reduced anxiety in practice. This finding is consistent with recent evidence that continuing professional development and organizational support for learning are relevant to nurse retention, engagement, and well-being [39,40]. In public hospitals, professional development should be accessible to nurses across departments and career stages, particularly those working in high-demand units. Training should not be limited to technical skills but should also include communication, leadership, psychological safety, conflict management, and occupational health.

The mixed-methods design is a major strength of this study. The quantitative phase identified which dimensions of work stress and quality of work life were associated with burnout, while the qualitative phase explained why these factors mattered in daily nursing practice. The integration of findings showed that burnout appeared to be related to the combination of high job demands and insufficient workplace resources. This is especially useful for occupational health nursing because it points to practical intervention targets: staffing adequacy, fair scheduling, compensation, professional development, psychological safety, organizational justice, and supportive leadership. The findings are also relevant to the broader public health workforce agenda because nurse burnout may contribute to turnover intention, absenteeism, reduced productivity, and compromised patient care [3,25,41]. In this sense, improving nurses’ quality of work life is both a workforce protection strategy and a patient safety strategy.

The findings of this study are generally consistent with international evidence showing that workload and insufficient staffing are associated with nurse burnout [10,22], while organizational climate, work–life imbalance, and limited workplace resources are also important workplace factors related to burnout and nurse well-being [5,8,11]. However, the present study adds context-specific evidence from provincial public hospitals in Thailand, where nurses may experience burnout within a public service environment shaped by staffing constraints, extended working hours, and organizational policies. In the present study, the finding that work–life balance had the strongest association with burnout may reflect the high proportion of participants who reported working more than 10 h per day. In contrast, the unexpected negative coefficient for interpersonal relationship-related stress should be interpreted cautiously because it may reflect shared variance among psychosocial workplace variables in the multivariable model. These findings suggest that future research should use larger samples and adjusted models to examine whether these associations remain stable across different hospital settings.

These findings have several implications for practice and policy. First, hospital administrators should routinely assess burnout, work stress, and quality of work life as part of occupational health surveillance. Second, nurse managers should prioritize workload management, staffing adequacy, and fair shift scheduling to protect recovery time and reduce cumulative fatigue. Third, organizational policies should strengthen fairness, communication, participation in decision-making, and recognition of nursing work. Fourth, professional development should be embedded into workforce planning and made accessible to nurses across units. Finally, occupational health nursing interventions should combine individual support with organizational change. Mindfulness, counseling, or stress management may be helpful, but they are unlikely to be sufficient if staffing shortages, long working hours, and unsupportive organizational climates remain unchanged [26,27,42].

### Limitations

This study has limitations. First, the quantitative phase used a cross-sectional design, which limits causal interpretation. Although several dimensions of work stress and quality of work life were associated with burnout, the direction of these associations cannot be confirmed. Second, the achieved quantitative sample was smaller than the calculated sample size. Although the participant-to-predictor ratio was considered acceptable for the planned multiple regression analysis, the reduced sample size may have limited the ability to detect smaller predictor-specific effects. Third, data were collected from registered nurses working in public hospitals in Samut Songkhram Province, Thailand. Therefore, the findings should be interpreted within this provincial public hospital context and should not be generalized to all nurses in Thailand, private hospitals, tertiary medical centers, or public hospitals in other regions. Fourth, the quantitative variables were collected using the same self-report questionnaire at a single time point, which may introduce common method bias. The observed associations among work stress, quality of work life, and burnout may partly reflect shared measurement method rather than only substantive relationships among the constructs. Fifth, demographic and work-related characteristics were not included as covariates in the primary regression model. Although this approach was consistent with the study objective of examining work stress and quality of work life dimensions, the findings may differ if age, marital status, department, years of service, working hours, or other individual and occupational characteristics are included in adjusted models. Sixth, the sample was highly gender-imbalanced, as 185 participants were female and only one participant was male. Therefore, the findings primarily reflect the experiences of female registered nurses working in the participating public hospitals and may not fully represent male nurses or more gender-diverse nursing populations. Burnout, work–life balance, and occupational stress may also be shaped by gender-related family, work, and social expectations. Seventh, the qualitative phase included 10 participants, which provided contextual depth but may not capture the full diversity of nurses’ experiences across all units and career stages. Eighth, the regression analysis used an aggregate burnout score as the dependent variable. Although this approach provided a parsimonious summary of overall burnout risk, burnout is a multidimensional construct, and aggregation may obscure dimension-specific relationships between emotional exhaustion, depersonalization or cynicism, reduced personal accomplishment, and workplace factors. Finally, although the model explained 35.0% of the variance in burnout, other relevant factors—such as resilience, coping style, leadership style, workplace violence, patient acuity, staffing ratios, and family responsibilities—were not included and should be examined in future research.

Future studies should consider longitudinal or multicenter designs to clarify the temporal relationships among work stress, quality of work life, and burnout. Research should also examine organizational interventions targeting work–life balance, staffing adequacy, scheduling flexibility, compensation fairness, peer support, and leadership practices. Future mixed-methods studies should continue to use participant quotations and joint displays to show more explicitly how qualitative findings explain quantitative results.

## 5. Conclusions

This explanatory sequential mixed-methods study found that burnout among registered nurses working in public hospitals in Samut Songkhram Province, Thailand, was associated with both work stress and quality of work life. A large proportion of nurses reported high work stress and were at risk of burnout. Work stress and quality of work life jointly explained 35.0% of the variance in burnout. Work–life balance, fair and adequate compensation, opportunities for professional development, organizational justice, safe working conditions, and social integration in the workplace were associated with lower burnout scores. The qualitative findings explained and expanded these quantitative results by showing how workload, staffing shortages, extended working hours, limited resources, and insufficient recovery time shaped nurses’ experiences of stress and burnout, while supervisor support, peer relationships, fairness, and professional development were described as important workplace resources. These findings identify modifiable organizational factors associated with burnout that may inform future workplace interventions, nursing management strategies, and occupational health nursing programs aimed at improving nurses’ quality of work life and supporting public hospital workforce sustainability in this provincial context.

## Figures and Tables

**Table 1:** Demographic and work-related characteristics of registered nurses.

Characteristics	n	%
**Sex**		
Female	185	99.46
Male	1	0.54
**Age group, years**		
21–30	63	33.87
31–40	53	28.49
41–50	38	20.43
51–60	32	17.20
**Marital status**		
Single	84	45.16
Married/living with spouse	89	47.85
Divorced/widowed/separated	13	6.99
**Educational level**		
Bachelor’s degree	182	97.85
Master’s degree	4	2.15
**Professional position**		
Registered nurse, operational level	58	31.18
Registered nurse, practitioner level	108	58.06
Registered nurse, senior practitioner level	15	8.06
Registered nurse, expert/senior expert level	3	1.61
Head nurse/nursing administrator	2	1.08
**Monthly income, Thai Baht**		
<20,000	18	9.68
20,001–30,000	60	32.26
30,001–40,000	44	23.66
40,001–50,000	24	12.90
>50,000	40	21.51
**Department/unit**		
Outpatient department	27	14.52
Inpatient department	79	42.47
Labor room/operating room/emergency department	34	18.28
Specialized units, e.g., ICU/CCU	43	23.12
Nursing administration	3	1.61
**Years of government service**		
<5 years	34	18.28
5–10 years	54	29.03
11–15 years	34	18.28
16–20 years	10	5.38
>20 years	54	29.03
**Average working hours per day**		
8 h	31	16.67
9 h	9	4.84
10 h	13	6.99
>10 h	133	71.51

Note. Percentages were calculated based on the total sample size of 186 registered nurses. Percentages may not total exactly 100.00 because of rounding. Head nurse and nursing administration participants were included only if they retained direct nursing practice, supervisory, or unit-based clinical responsibilities. ICU = intensive care unit; CCU = coronary care unit.

**Table 2:** Overall levels of work stress, quality of work life, and burnout among registered nurses.

Variable and Level	Score Range	n	%
**Work stress**			
High	3.68–5.00	123	66.13
Moderate	2.34–3.67	63	33.87
Low	1.00–2.33	0	0.00
**Quality of work life**			
Very good	4.21–5.00	49	26.34
Good	3.41–4.20	74	39.78
Moderate	2.61–3.40	49	26.34
Poor	1.81–2.60	14	7.53
Very poor	1.00–1.80	0	0.00
**Burnout**			
No burnout risk	0.00–1.20	2	1.08
Low burnout risk	1.21–2.40	17	9.14
Moderate burnout risk	2.41–3.60	49	26.34
At risk of burnout	3.61–4.80	113	60.75
Burnout	4.81–6.00	5	2.69

**Table 3:** Mean scores of work stress, quality of work life, and burnout dimensions among registered nurses.

Variable/Dimension	Mean	SD	Interpretation
**Work stress**			
Job characteristics	3.53	0.48	Moderate
Career achievement and advancement	3.51	0.54	Moderate
Interpersonal relationships	3.62	0.56	Moderate
Organizational structure, policy, and climate	3.50	0.50	Moderate
**Overall work stress**	**3.54**	**0.52**	**Moderate**
**Quality of work life**			
Fair and adequate compensation	3.78	0.65	High
Safe and healthy working conditions	3.92	0.71	High
Career security and advancement	3.77	0.73	High
Opportunities for knowledge and skill development	4.00	0.71	High
Social integration in the workplace	3.92	0.64	High
Fairness and respect for individual rights	4.07	0.70	High
Work–life balance	3.90	0.79	High
Social responsibility	4.21	0.71	High
**Overall quality of work life**	**4.07**	**0.61**	**High**
**Burnout**			
Emotional exhaustion	4.23	1.36	At risk of burnout
Depersonalization/cynicism	4.43	1.16	At risk of burnout
Reduced personal accomplishment	2.36	1.13	Low burnout risk
**Overall burnout**	**3.67**	**1.22**	**At risk of burnout**

**Table 4:** Multiple regression analysis predicting burnout among registered nurses.

Predictor	B	SE	β	t	*p*-Value
Constant	3.44	0.43	—	7.99	<0.001
**Work stress dimensions**					
Job characteristics	−0.13	0.19	−0.09	−0.67	0.50
Career achievement and advancement	0.15	0.17	0.11	0.93	0.36
Interpersonal relationships	−0.32	0.16	−0.24	−2.00	0.04
Organizational structure, policy, and climate	0.34	0.17	0.23	2.00	0.04
**Quality of work life dimensions**					
Fair and adequate compensation	−0.41	0.14	−0.31	−2.93	0.004
Safe and healthy working conditions	−0.28	0.13	−0.22	−2.15	0.03
Career security and advancement	−0.19	0.15	−0.14	−1.27	0.20
Opportunities for knowledge and skill development	−0.36	0.16	−0.27	−2.25	0.02
Social integration in the workplace	−0.25	0.12	−0.18	−2.07	0.04
Fairness and respect for individual rights	−0.30	0.13	−0.26	−2.31	0.02
Work–life balance	−0.47	0.15	−0.35	−3.13	0.002
Social responsibility	−0.11	0.12	−0.08	−0.92	0.35

Note. Dependent variable: burnout. B = unstandardized regression coefficient; SE = standard error; β = standardized regression coefficient. Model statistics: R^2^ = 0.350, adjusted R^2^ = 0.305, F(12, 173) = 7.76, *p* < 0.001.

**Table 5:** Qualitative themes explaining work stress, quality of work life, and burnout among registered nurses.

Theme	Description	Link to Quantitative Findings
Workload and increased responsibilities	Nurses reported heavy workload, staffing shortages, high patient volume, and frequent urgent clinical situations as major sources of stress and fatigue.	Supports the high proportion of nurses reporting high work stress and burnout risk.
Work–life imbalance	Shift work, overtime, and insufficient rest limited nurses’ time for family, recovery, and personal activities.	Helps explain why work–life balance was the workplace factor most strongly associated with lower burnout scores in the regression model.
Organizational and peer support	Support from supervisors, colleagues, fair treatment, and positive workplace relationships helped nurses cope with stress.	Supports the relevance of organizational justice, peer and supervisor support, and organizational climate in understanding nurses’ burnout experiences.
Professional development and self-care	Nurses viewed skill development, confidence in practice, rest, exercise, and emotional support as important coping resources.	Explains why opportunities for knowledge and skill development were negatively associated with burnout.

**Table 6:** Joint display integrating quantitative and qualitative findings.

Quantitative Finding	Qualitative Explanation	Integrated Interpretation
Most nurses were classified as having high work stress, although the overall mean score was moderate.	Nurses described heavy workload, staffing shortages, high patient volume, and urgent clinical situations.	Work stress was common in daily practice, especially when staffing and workload pressures limited recovery time.
Work–life balance was associated with lower burnout scores.	Nurses reported that rotating shifts, overtime, and insufficient rest limited time for family, recovery, and personal activities.	Work–life balance appeared to be an important workplace resource related to lower burnout.
Organizational structure, policy, and climate were associated with higher burnout scores.	Nurses described organizational constraints, limited flexibility, and the need for supportive leadership.	Organizational climate may intensify or buffer burnout depending on how policies and support are experienced.
Fair compensation, workplace safety, organizational justice, social integration, and professional development were associated with lower burnout scores.	Nurses emphasized fair treatment, peer and supervisor support, safe working conditions, and opportunities for skill development.	Quality of work life dimensions functioned as job resources that may support nurses’ well-being and reduce burnout risk.

Note. The joint display shows how qualitative themes explained and expanded the quantitative results in the explanatory sequential mixed-methods design.

## Data Availability

The data presented in this study are available from the corresponding author upon reasonable request. The data are not publicly available due to privacy and ethical restrictions related to participant confidentiality.

## Abbreviations

The following abbreviations are used in this manuscript:

CCU	Coronary Care Unit
ICU	Intensive Care Unit
